# Weighed Gene Coexpression Network Analysis Screens the Potential Long Noncoding RNAs and Genes Associated with Progression of Coronary Artery Disease

**DOI:** 10.1155/2020/8183420

**Published:** 2020-07-06

**Authors:** Lang Wang, Jun Hu, Jiali Zhou, Fan Guo, Tan Yao, Liang Zhang

**Affiliations:** ^1^Department of Cardiology, Renmin Hospital of Wuhan University, Wuhan 430060, China; ^2^Cardiovascular Research Institute, Wuhan University, Wuhan 430060, China; ^3^Hubei Key Laboratory of Cardiology, Wuhan 430060, China; ^4^Department of Cardiology, Huangzhou District People's Holpital, Huanggang 438000, China; ^5^Department of Cardiology, Wuhan Fifth Hospital, Wuhan 438000, China; ^6^Department of Cardiology, Luotian Wanmizhai Hospital, Huanggang 438600, China

## Abstract

**Background:**

Coronary artery disease (CAD) is a type of heart disease with a high morbidity rate. This study is aimed at identifying potential biomarkers closely related to the progression of CAD.

**Materials and Methods:**

A microarray dataset of GSE59867 was downloaded from a public database, Gene Expression Omnibus, which included 46 cases of stable CAD without a history of myocardial infarction (MI), 30 cases of MI without heart failure (HF), and 34 cases of MI with HF. Differentially expressed long noncoding RNAs (DElncRNAs) and mRNAs (DEmRNAs) were identified by the limma package, and functions of DEmRNAs were annotated by Gene Ontology and KEGG pathways. In addition, weighed gene coexpression network analysis (WGCNA) was used to construct a coexpression network of DEmRNAs, and a disease-related lncRNAs-mRNAs-pathway network was constructed. Finally, the datasets of GSE61145 and GSE57338 were used to verify the expression levels of the above highly correlated candidates.

**Results:**

A total of 2362 upregulated mRNAs and 2816 downregulated mRNAs, as well as 235 upregulated lncRNAs and 113 downregulated lncRNAs were screened. These genes were significantly enriched in “cytokine-cytokine receptor interaction,” “RIG-I-like receptor signaling pathway,” and “natural killer cell-mediated cytotoxicity.” Five modules including 1201 DEmRNAs were enriched in WGCNA. A coexpression network including 19 DElncRNAs and 413 DEmRNAs was constructed. These genes were significantly enriched in “phosphatidylinositol signaling system,” “insulin signaling pathway,” and “MAPK signaling pathway”. Disease-related gene-pathway network suggested *FASN* in “insulin signaling pathway,” *DGKZ* in “phosphatidylinositol signaling system,” and *TNFRSF1A* in “MAPK signaling pathway” were involved in MI.

**Conclusion:**

*FASN*, *DGKZ*, and *TNFRSF1A* were revealed to be CAD progression-associated genes by WGCNA coexpression network analysis.

## 1. Introduction

Coronary artery disease (CAD) is the most common type of cardiovascular event [1]. The mortality of CAD has been increased from 5.74 million deaths at 1990 to 8.14 million deaths at 2013 [2]. Metabolic syndrome such as insulin resistance/glucose intolerance (diabetes), high blood pressure, and obesity would significantly exacerbate the disease [1]. Myocardial infarction (MI) is a type of the complications of CAD with a high morbidity rate and a mortality rate of 5% [3]. Acute MI induces left ventricular remodeling, which is a predictive sign for a future heart failure (HF). CAD and its complications remain the number one cause of hospital admission diagnosis in elderly patients [4].

Though considerable efforts have been made during the past decades, the pathophysiologic mechanisms in the development and progression of MI and HF remain elusive and require further investigation. Molecular biology studies have demonstrated that several genes are associated with the development and progression of CAD [5]. *TREML4* (triggering receptor expressed on myeloid cell-like 4), which was upregulated in coronary artery calcification, was reported to be involved in the formation of calcified atheromatous plaque [6]. Some genes, such as *GLO1* (glyoxalase I) and *PPIL1* (peptidylprolyl isomerase I), play important regulatory roles in CAD risk processes including metabolism, signal transduction, coagulation, immunity, and proteolysis [7]. In addition, Maciejak et al. [8] used microarrays to analyze differentially expressed mRNA in HF and identified a set of genes including *FMN1*, *JDP2*, and *RNASE1*, which were transcriptomic biomarkers of HF development. The candidate genes involved in the molecular mechanism of CAD with clinical implications would be used as potential novel biomarkers and targets for therapeutic intervention of CAD. However, no effective genes could be used in a clinical setting currently, and the cellular mechanisms of genes involved in the pathological process of CAD remain largely unexplained.

In the current study, a microarray dataset was downloaded from the public database of Gene Expression Omnibus (GEO, http://www.ncbi.nlm.nih.gov/geo) and was reanalyzed using the widely used bioinformatics methods to identify potential genes related with CAD progression. This study may help to reveal the relationship between candidate genes with altered expression and the development and progression of CAD.

## 2. Materials and Methods

### 2.1. Data Source

The gene expression dataset of GSE59867 [8] was downloaded from GEO database. This dataset included gene expression profiles derived from the peripheral blood of 390 MI cases and 46 stable CAD patients without a history of MI. We selected gene expression data of 30 MI patients without HF, 34 MI patients with HF, and 46 stable CAD patients. Besides, the samples of 30 MI without HF and 34 MI with HF were further divided into 4 groups with different times: 1 day after MI, 4-6 days after MI, 1 month after MI, and 6 months after MI. This dataset was based on the platform GPL6244 Affymetrix Human Gene 1.0 ST Array. In addition, GSE61145 [9] and GSE57338 [10] were also obtained from GEO and used as the validation datasets. Seven normal samples and seven ST-elevation MI (STEMI) samples were chosen from the dataset of GSE61145, while 136 individuals with normal hearts and 95 patients with HF were selected from the dataset of GSE57338.

### 2.2. Identification of Differentially Expressed Genes (DEGs)

The original data GSE59867 (.txt) were downloaded and log_2_ transformed using the limma package (version: 3.32.5) [11] in R3.4.1 for normalizing the microarray data. The platform annotation files, transcript IDs, and RefSeq ID were downloaded as previously described [12, 13]. The expression profiles were reannotated into mRNAs and lncRNAs by using the HUGO Gene Nomenclature Committee (HGNC) (http://www.genenames.org/) [14] which included 3979 lncRNAs and 19198 protein coding genes. Briefly, the reference genome of the human genome (GRCh38) provided by the HGNC database (https://www.gencodegenes.org/human/) was used to realign, and the unique alignment sequence remained. Meanwhile, based on the corresponding gtf gene annotation files, we kept annotation information for “protein-coding” genes as mRNA and genes of “antisense,” “sense-intronic,” “lincRNA,” “sense-overlapping,” “processed transcript,” “3prime–overlapping-ncRNA,” and “noncoding” as lncRNA. After removing the unmatched probes of gene symbol, 595 lncRNAs and 17790 mRNAs were obtained.

All samples were divided into three groups: stable CAD samples, MI without HF, and MI with HF. The limma package in R 3.4.1 [15] was used to screen the differentially expressed lncRNAs (DElncRNAs) and DEmRNAs in three pairwise comparisons. The FDR (false discovery rate) < 0.05 and ∣log fold change (FC) | >0.5 were used as the cut-off criteria. Bidirectional hierarchical clustering was performed by using pheatmap (version 1.0.8) (https://cran.r-project.org/package=pheatmap) in R 3.4.1. Further, the DEmRNAs were used to perform Gene Ontology (GO) and KEGG pathway enrichment analyses using DAVID 6.8 (https://david.ncifcrf.gov/) [16, 17].

### 2.3. Coexpression Network Construction by WGCNA

A coexpression network was built using weighed gene coexpression network analysis (WGCNA) package (version 1.61) in R [18]. The overlapped differentially expressed genes (DEGs) between each pairwise comparison were submitted for WGCNA analysis to screen modules significantly associated with disease status and time points. The parameters were set as (1) more than or equal to 100 DEGs included in one module, (2) cutHeight = 0.95, and (3) *P* < 0.05. A WGCNA map was constructed which divided the DEGs into several modules based on the above analysis. For the lncRNAs and mRNAs in the modules screened from the previous step, cor function (http://77.66.12.57/R-help/cor.test.html) in R was utilized to calculate the Pearson correlation coefficient (PCC) among their expression levels. A coexpression network was then built and visualized by Cytoscape 3.6.1 [19] (http://www.cytoscape.org/). The genes in the coexpression network were submitted for pathway enrichment analysis using DAVID.

### 2.4. Construction of Disease-Related lncRNA-mRNA-Pathway Network

The MI- or HF-related KEGG pathways and genes were searched in the Comparative Toxicogenomics Database (CTD) 2017 update (http://ctdbase.org/) by searching words of “heart failure” or “myocardial infarction”. The resulting pathways and genes were compared with those pathways and genes in the coexpression network. The overlapped pathways and genes were used to construct a disease-related gene and pathway network. Finally, the expression levels of important genes in the network were plotted at different times (1 d (admission), 4-6 d (discharge), and 1 m and 6 m (stable phase)) under different disease representations.

### 2.5. Statistical Analysis

R packages (V 3.4.1, University of Auckland, Auckland, New Zealand) were used to statistically analyze. *P* < 0.05 was considered statistically significant.

## 3. Results

### 3.1. Identification of DEGs among the Three Groups

**Figure 1:**
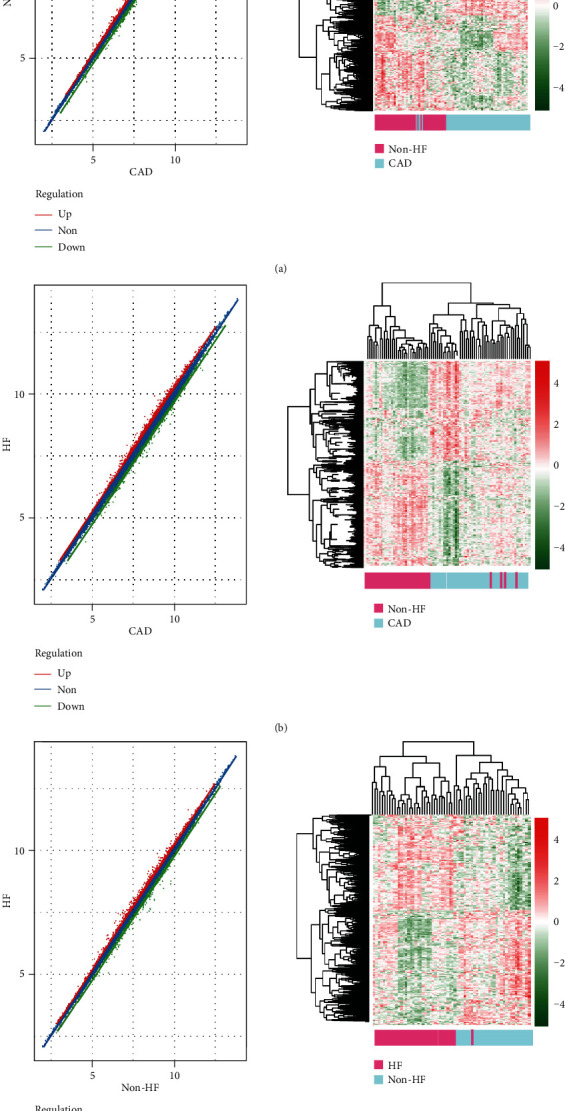
The scatter distribution map and bidirectional hierarchical clustering map based on DEG expression level in the CAD vs. MI without HF group (a), CAD vs. MI with HF group (b), and MI with HF vs. MI without HF group (c).

**Figure 2:**
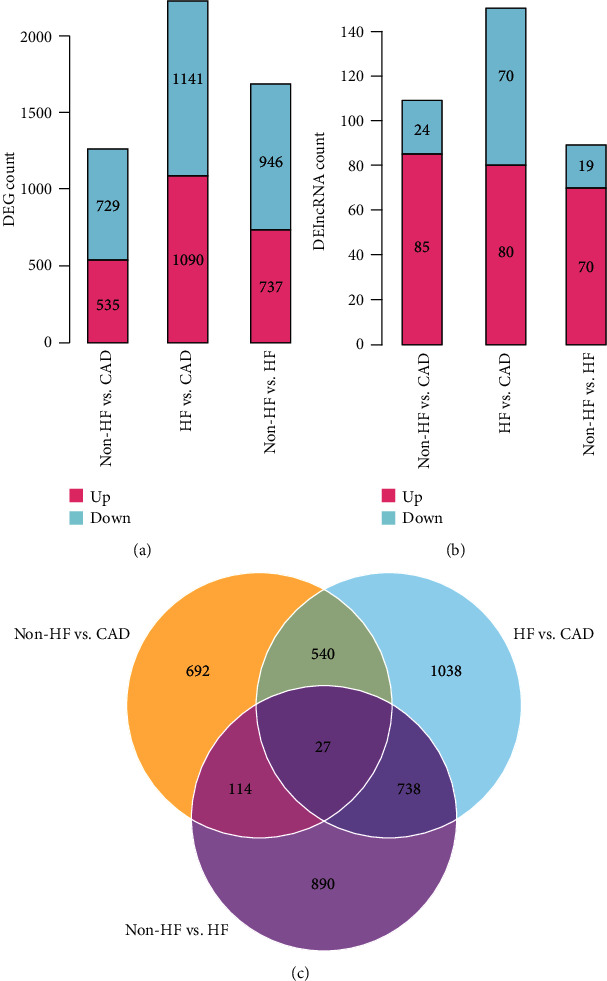
The DEGs (a) and DElncRNAs (b) in each comparison showed significant differences in the direction distribution of the histogram. (c) The Venn diagram was compared between the two groups.

The dataset GSE59867 was downloaded from the GEO database, and DEGs between stable CAD patients and MI patients without HF, stable CAD patients and MI patients with HF, and MI patients without HF and MI patients with HF were identified using the limma package. The number of DEmRNAs and DElncRNAs was displayed in Figure 1. Totally, 1264 DEmRNAs and 109 DElncRNAs between stable CAD patients and MI patients without HF, 2231 DEmRNAs and 150 DElncRNAs between stable CAD patients and MI patients with HF, and 1683 DEmRNAs and 89 DElncRNAs between MI patients without HF and MI patients with HF were identified. A total of 4039 DEGs were overlapped among the three comparisons. The scatter distribution and hierarchical clustering map of DEGs in each pairwise comparison were displayed in Figure 2. From these results, the DEGs in each comparison can separate the samples in different groups obviously, suggesting that the DEGs are sample characteristic and credible.

### 3.2. Functional Enrichment Analysis of DEGs

**Figure 3:**
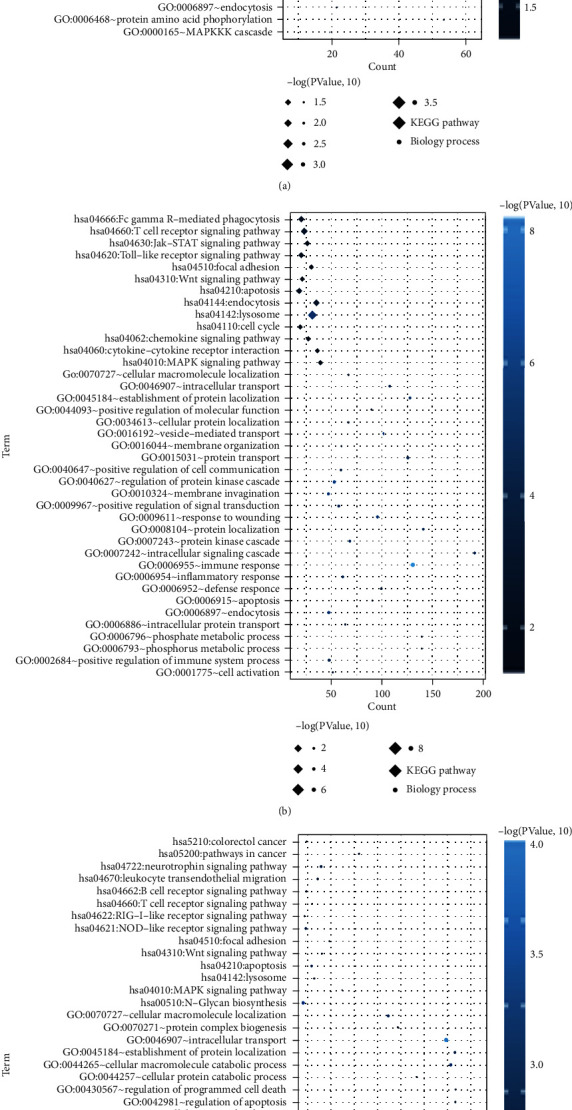
The DEGs significantly associated with BP and KEGG signaling pathways. The CAD vs. MI without HF group (a). The CAD vs. MI with HF group (b). The MI with HF vs. MI without HF group (c). The horizontal axis represents the number of DEGs, the vertical axis represents the name, the diamond and the circle represent the BP and KEGG paths, respectively, and the color of the dots represents the significant *P* value, and the darker the color, the higher the significance.

The DEmRNAs were submitted to GO and KEGG pathway enrichment analyses further. Twenty-four, 26, and 23 GO biological processes and 13, 13, and 14 KEGG pathways, respectively, were enriched by the DEmRNAs between the three comparisons. As shown in Figure 3, the DEmRNAs between stable CAD patients and MI patients without HF were enriched into pathways including “calcium signaling pathway,” “Jak-STAT signaling pathway,” “neuroactive ligand-receptor interaction,” and “RIG-I-like receptor signaling pathway” as well as biological processes of “MAPKKK cascade,” “cell cycle,” and “positive regulation of cell proliferation.” The DEGs between stable CAD patients and MI patients with HF were enriched into pathways including “MAPK signaling pathway,” “cytokine-cytokine receptor interaction,” “cell cycle,” and “focal adhesion” as well as biological processes such as “phosphorus metabolic process,” “apoptosis,” and “defense response.”. The DEGs between MI patients with HF and MI patients without HF were enriched into pathways including “pathways in cancer,” “MAPK signaling pathway,” and “focal adhesion” as well as biological processes such as “regulation of programmed cell death,” “regulation of apoptosis,” and “regulation of cell death.”.

### 3.3. Disease-Related Modules Identified by WGCNA

**Figure 4:**
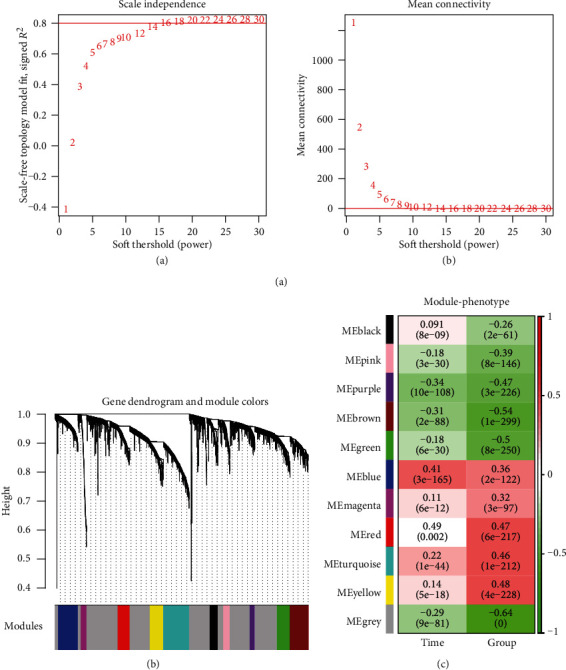
The WGCNA modules. (a, A) Adjacency matrix weight parameter power selection graph. The horizontal axis represents the weight parameter power, and the vertical axis represents the square of the log(*k*) and log(*p*(*k*)) correlation coefficients in the corresponding network. The red line indicates the standard line where the square of the correlation coefficient reaches 0.9. (B) Schematic diagram of the average connectivity of RNA under different power parameters. The red line indicates the value of the average connectivity of the network nodes [1] under the value of the power parameter of the A matrix adjacency matrix. (b) The module divides the tree, each color representing a different module. (c) Correlation heatmap between the module and sample representation.

The 4039 overlapped DEGs were submitted to WGCNA analysis. To qualify the scale-free network distribution, the minimum power value was set as the value when the squared correlation coefficients first reached 0.8. As shown in Figure 4(a), the power value is 16 (Figure 4(a)). At this point, the average degree of the coexpression network is 1, which is completely in conformity with small-world network properties. A hierarchical cluster tree including 10 modules was obtained (Figure 4(b)). The correlations between each module and sample characterizations were calculated. Results showed that blue, magenta, red, turquoise, and yellow modules were significantly positively correlated with disease progression and time points (Figure 4(c)). The 1201 DEGs including 83 DElncRNAs and 1119 DEmRNAs were submitted for further study.

### 3.4. Construction of Coexpression Network

**Figure 5:**
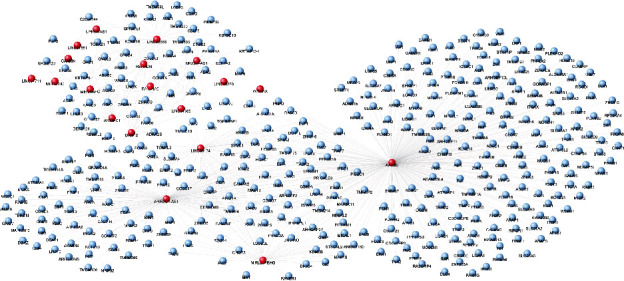
The lncRNA-mRNA coexpression network. The red and blue nodes represent lncRNAs and mRNAs, respectively.

The PCCs among the 1201 DEGs were calculated, and the relationships with PCC > 0.7 were retained. A coexpression network was built using these relationships (Figure 5). This network was composed of 19 DElncRNAs and 413 DEmRNAs. KEGG pathway annotation was performed for the nodes in the coexpression network. A total of 13 pathways were significantly enriched including “phosphatidylinositol signaling system,” “Jak-STAT signaling pathway,” “neurotrophin signaling pathway,” “MAPK signaling pathway,” and “focal adhesion”.

### 3.5. Construction of Disease-Related lncRNA-mRNA-Pathway Network

**Figure 6:**
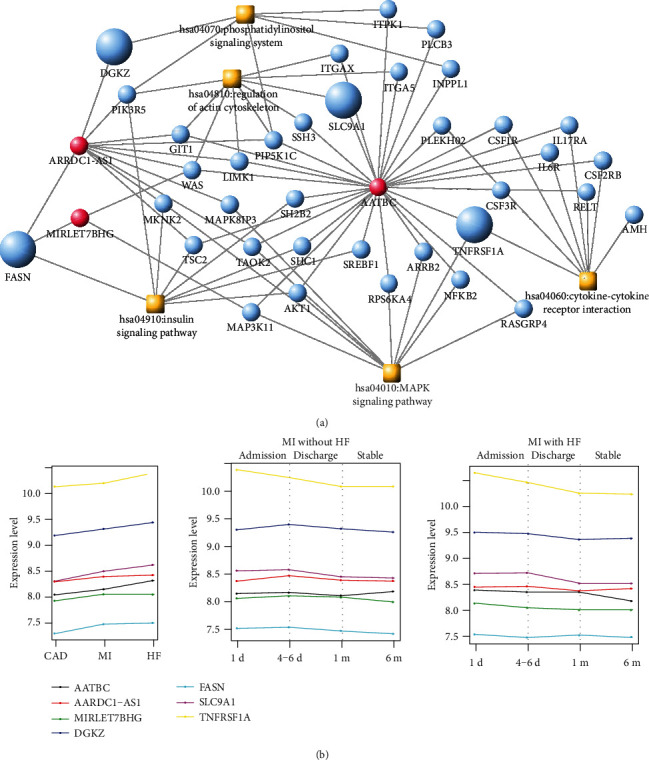
(a) Disease-associated lncRNA-mRNA-pathway network. The red and blue dots indicate lncRNAs and mRNAs, respectively, the orange square indicates the disease pathway, and the enlarged node indicates the disease gene. (b) The expression levels of four disease genes and three coexpressed lncRNAs in different phases (CAD, MI without HF, and MI with HF) and at different times (1 d (admission), 4-6 d (discharge), and 1 m and 6 m (stable)) in the same disease phase. Different colors represent different genes.

A total of 201 HF-related KEGG pathways and 104 HF-related genes as well as 190 MI-related KEGGs and MI-related genes were obtained from CTD. Five KEGG pathways including 4 disease genes related to both HF and MI were extracted. A disease-related lncRNA-mRNA-pathway network was built (Figure 6(a)). This network consisted of 3 lncRNAs (ARRDC1-AS1, AATBC, and MIRLET7BHG), 5 disease-related pathways (hsa04910: insulin signaling pathway, hsa04010: MAPK signaling pathway, hsa04060: cytokine-cytokine receptor interaction, hsa04810: regulation of actin cytoskeleton, and hsa04070: phosphatidylinositol signaling system), 4 disease-related mRNAs (*DGKZ*, *SLC9A1*, *TNFRSF1A*, and *FASN*), and 32 genes in the disease pathways. From this network, the disease gene *FASN* was involved in the “insulin signaling pathway,” *DGKZ* was involved in the “phosphatidylinositol signaling system,” *SLC9A1* was involved in “regulation of actin cytoskeleton,” and *TNFRSF1A* was involved in the “MAPK signaling pathway.” The lncRNA *ARRDC1-AS1* might coexpress with *FASN* and *DGKZ* to be involved in the “insulin signaling pathway” and “phosphatidylinositol signaling system.” The lncRNA *AATBC* might coexpress with *TNFRSF1A* to be involved in “cytokine-cytokine receptor interaction.” The lncRNA *MIRLET7BHG* might coexpress with *FASN* to be involved in the “insulin signaling pathway.” These lncRNAs and pathways might be closely related with the progression of CAD.

The changes in the expression levels of four disease genes and three coexpressed lncRNAs in different phases (CAD, MI without HF and MI with HF) and at different times in the same disease phase were shown in Figure 6(b). It was found that the expression level of *TNFRSF1A* was the highest both in different phases and at different times in the same disease phase, while the expression level of *FASN* was the lowest compared with the other genes (Figure 6(b)).

### 3.6. Validation of the Candidate Genes

**Figure 7:**
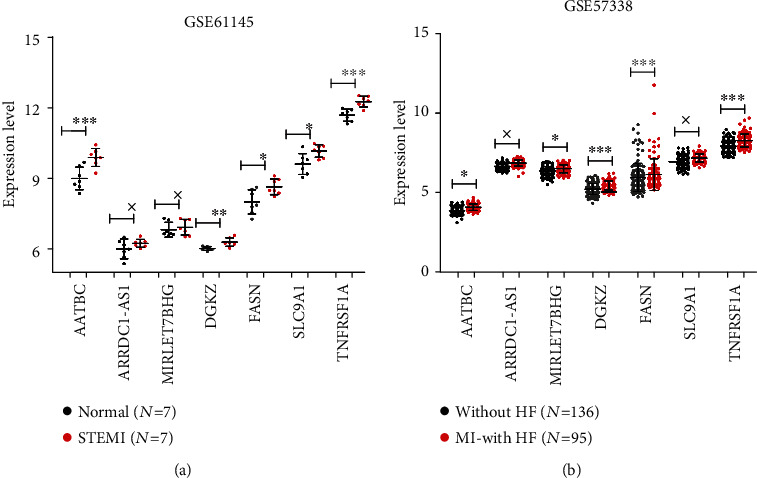
The expression levels of four disease mRNAs (*DGKZ*, *SLC9A1*, *TNFRSF1A*, and *FASN*) and three coexpressed lncRNAs (*ARRDC1-AS1*, *AATBC*, and *MIRLET7BHG*) were verified in the validation datasets (GSE61145 and GSE57338). ^×^*P* > 0.05, compared with the normal or without HF. ^∗^*P* < 0.05 or ^∗∗∗^*P* < 0.01, compared with the normal or without HF.

The expression levels of these closely related genes, including three lncRNAs *ARRDC1-AS1*, *AATBC*, and *MIRLET7BHG*, as well as four mRNAs *DGKZ*, *SLC9A1*, *TNFRSF1A*, and *FASN* were verified in the validation datasets (GSE61145 and GSE57338). The results showed that in the GSE61145 dataset, the expression levels of *AATBC*, *DGKZ*, *FASN*, *SLC9A1*, and *TNFRSF1A* were significantly upregulated in the STEMI group, compared with the normal group (*P* < 0.05, Figure 7(a)). In the GSE57338 dataset, the expression levels of *AATBC*, *MIRLET7BHG*, *DGKZ*, *FASN*, and *TNFRSF1A* were significantly upregulated in the patients with HF group, compared with the normal group (*P* < 0.05, Figure 7(b)). These results indicated that the consistency rate for the validation datasets and the training dataset was 85.71%. This reveals that the four disease mRNAs and their coexpressed lncRNAs may be acted as the potential candidates associated with the progression of HF in CAD.

## 4. Discussion

CAD has become a major health concern in the last few decades; therefore, future studies need to concentrate on controlling CAD risk factors [20]. In the present study, the dataset of GSE59867 was downloaded from the GEO database to analyze DEGs associated with the development of CAD. Totally, 1264 DEmRNAs and 109 DElncRNAs between stable CAD patients and MI patients without HF, 2231 DEmRNAs and 150 DElncRNAs between stable CAD patients and MI patients with HF, and 1683 DEmRNAs and 89 DElncRNAs between MI patients without HF and MI patients with HF were identified. A DElncRNA-DEmRNA coexpression network was built by WGCNA, and KEGG analysis suggested that these genes were significantly enriched in 13 pathways. Finally, four disease mRNAs (*DGKZ*, *SLC9A1*, *TNFRSF1A*, and *FASN*) and three coexpressed lncRNAs (*ARRDC1-AS1*, *AATBC*, and *MIRLET7BHG*) were identified as the potential candidates in the development of CAD. The differential expressions of the seven genes were successfully validated by the independent datasets of GSE61145 and GSE57338.

In this study, we used WGCNA to build a DElncRNA-DEmRNA coexpression network and analyzed the DEGs in this network. WGCNA, a bioinformatics application, describes the correlation patterns between gene chip samples and provides direct biologically functional interpretations of gene network modules [18]. It has been successfully used to build the gene coexpression network of various diseases to explore their potential biomarkers [21]. In fact, the microarray dataset of GSE59867 was not analyzed by WGCNA first in this study. Mo et al. [22] used the dataset of GSE59867 as the validation dataset to verify the four gene signatures (*NCF2*, *MYO1F*, *S1PR4*, and *FCN1*) identified by WGCNA in GSE90074. A recent study by Niu et al. identified 6 hub genes (*BCL3*, *HCK*, *PPIF*, *S100A9*, *SERPINA1*, and *TBC1D9B*) by analyzing GSE59867 using WGCNA [23]. Though using the same dataset and similar analysis method, our study was different from their study. The input of WGCNA was the genes exhibiting the top 50% in high expression variance. In our study, the input of WGCNA was the 4039 overlapped DEGs. Therefore, the lncRNAs and mRNAs in the coexpression network were differentially expressed during CAD development.

In the disease-related lncRNA-mRNA-pathway network, *FASN* was involved in the “insulin signaling pathway,” *DGKZ* was involved in the “phosphatidylinositol signaling system,” and *TNFRSF1A* was involved in the “MAPK signaling pathway.” Fatty acid synthase (*FASN*) is the sole cytosolic mammalian enzyme for de novo lipid synthesis. One of the features of cancer cells is the increased de novo lipogenesis, and *FASN* is part of the metabolic reprogramming cancer hallmark [24–26]. The expression of *FASN* is significantly upregulated in many cancer types while it is extremely low in nonmalignant tissues [27]. Numerous studies have reported its importance for cancer cell survival and its association with poor prognosis. For example, Wu et al. demonstrated that *FASN* could suppress the expression of *NF-κB* but increase the expression of specificity protein 1 and regulate DNA repair to increase survival against genotoxic insults [28]. *FASN* has received much attention as a cancer therapeutic target [24]. Several FASN inhibitors have been developed for cancer therapy during the past decades, such as orlistat [29]. However, the expression of *FASN* in CAD has few been reported. Myocardial FA substrate metabolism is a feature of late-stage HF [30]. The protein level of FASN in cardiac tissue specimens of HF patients was significantly increased compared to those of control patients [31]. In this study, the lncRNAs *ARRDC1-AS1* and *MIRLET7BHG* could coexpress with *FASN* to be involved in “insulin signaling pathway.” The lncRNA *ARRDC1-AS1* was involved in a 9-lncRNA signature to predict recurrence of breast cancer [32]. However, *ARRDC1-AS1* was not reported in CAD previously. *MIRLET7BHG* is the miRNA let-7b host gene and has been previously reported to be implicated in metabolic disorders [33]. It was reported to be correlated with body mass index in polycystic ovary syndrome [34]. miRNA let-7B, transcribed from the lncRNA *MIRLET7BHG*, has been reported to be associated with cardiovascular diseases by many studies. hsa-let-7b was identified as a potential candidate regulator in acute MI [35, 36]. It was significantly upregulated in mobilized CD34+ progenitor cells in patients with segment-elevation MI [37]. Our results suggested that *ARRDC1-AS1* and *MIRLET7BHG* might have interacted with *FASN* to be involved in regulating insulin signaling pathways in patients with CAD.

*DGKZ* was involved in the “phosphatidylinositol signaling system,” and *TNFRSF1A* was involved in the “MAPK signaling pathway.” The PI3K/Akt signaling pathway is a critical pathway in the “phosphatidylinositol signaling system” and is a key signaling pathway involved in many life activities including cell division, differentiation, and apoptosis. PI3K/Akt signaling pathway was closely related to cardiovascular disease [38]. It was reported that insulin can protect cardiomyocytes from apoptosis by activating PI3K and Akt [39]. PI3K signaling is required for Exendin-4 to stimulate proliferation [40] and for oxidized low-density lipoprotein to promote angiogenesis in human coronary artery endothelial cells [41]. The PI3K family could be used as potential therapeutic targets for cardiovascular diseases including CAD [42]. In-depth studies of this series of related pathways and participating genes and lncRNAs have helped to identify more potent biomarkers of MI and HF.

The major limitation of this study is that the identified critical lncRNAs and genes were not verified on independent patients as well as *in vivo* or *in vitro* experiments. Future studies should be conducted to confirm the results of this study.

## 5. Conclusion

From the above, *FASN*, *DGKZ*, and *TNFRSF1A*, in “insulin signaling pathway,” “phosphatidylinositol signaling system,” and “MAPK signaling pathway”, would be potentially associated with the path mechanism of CAD. In addition, the cross-action effects among genetic and molecular processes would closely work in CAD. These three genes would be potentially represented by a novel molecular, mechanistic explanation for the pathologic basis of CAD and may be used as candidate targets for therapeutic discovery in CAD.

## Data Availability

The data used to support the findings of this study have been deposited in the gene expression omnibus repository with accession number of GSE59867.

## Additional Points

*Highlights*. (1) Dysregulated genes and lncRNAs between stable CAD, MI without HF, and MI with HF were identified. (2) A coexpression network included 19 DElncRNAs, and 413 DEmRNAs were constructed. (3) WGCNA identified that *FASN*, *DGKZ*, and *TNFRSF1A* were MI-associated genes.
